# Comprehensive analysis of liquid-liquid phase separation-related genes in prediction of breast cancer prognosis

**DOI:** 10.3389/fgene.2022.834471

**Published:** 2022-09-28

**Authors:** Huang Yu-Qing, Li Peng-Ping, Sun Ke, Yin Ke-Xing, Zhang Wei-Jun, Wang Zhen-Yu

**Affiliations:** ^1^ The First People’s Hospital of Xiaoshan, Hangzhou, China; ^2^ Xaioshan Hospital Affiliated to Wenzhou Medical University, Wenzhou, China

**Keywords:** liquid-liquid phase separation, multi-gene risk-score, prediction model, breast cancer, TCGA

## Abstract

**Objective:** Liquid-liquid phase separation (LLPS) is a functional unit formed by specific molecules. It lacks a membrane and has been reported to play a crucial role in tumor drug resistance and growth by regulating gene expression and drug distribution. However, whether LLPS could be used to predict cancer prognosis was not clear. This study aimed to construct a prognostic model for breast cancer based on LLPS-correlated genes (LCGs).

**Methods:** LCGs were identified using the PhaSepDB, gene expression profile and clinical characteristics of breast cancer were obtained from TCGA and cBioportal. The PanCancer Atlas (TCGA) cohort was used as the training cohort to construct the prognostic model, while the Nature 2012 and Nat Commun 2016 (TCGA) cohort and GEO data were used as test cohort to perform external verification. Data analysis was performed with R4.2.0 and SPSS20.0.

**Results:** We identified 140 prognosis-related LCGs (pLCGs) (*p*< 0.01) in all cohorts, 240 pLCGs (*p*< 0.01) in the luminal cohort, and 28 pLCGs (*p*< 0.05) in the triple-negative breast cancer (TNBC) cohort. Twelve genes in all cohorts (training cohort: 5/10-year ROC values were 0.76 and 0.77; verification cohort: 5/10-year ROC values were 0.61 and 0.58), eight genes in the luminal cohort (training cohort: 5/10-year ROC values were 0.79 and 0.75; verification cohort: 5/10-year ROC values were 0.62 and 0.62), and four genes in the TNBC cohort (training cohort: 5/10-year ROC values were 0.73 and 0.79; verification cohort: 5/10-year ROC values were 0.55 and 0.54) were screened out to construct the prognostic prediction model. The 17-gene risk-score was constructed in all cohorts (1/3/5-year ROC values were 0.88, 0.83, and 0.81), and the 11-gene risk-score was constructed in the luminal cohort (1/3/5-year ROC values were 0.67, 0.85 and 0.84), and the six-gene risk-score was constructed in the TNBC cohort (1/3/5-year ROC value were 0.87, 0.88 and 0.81). Finally, the risk-score and clinical factors were applied to construct nomograms in all cohorts (1/3/5-year ROC values were 0.89, 0.79 and 0.75, C-index = 0.784), in the luminal cohort (1/3/5-year ROC values were 0.84, 0.83 and 0.85, C-index = 0.803), and in the TNBC cohort (1/3/5-year ROC values were 0.95, 0.84 and 0.77, C-index = 0.847).

**Discussion:** This study explored the roles of LCGs in the prediction of breast cancer prognosis.

## Introduction

During the past decades, medical science has made obvious progress in the treatment of breast cancer, especially for *HER2*-positive breast cancer based on the development of *HER2-*targeted drugs, such as trastuzumab. However, because of the lack of useful gene targets, paclitaxel-centered combination chemotherapy was still the first-line treatment strategy for patients with *HER2*-negative breast carcinoma, including luminal and triple-negative breast cancer (TNBC), the resistance of which made for a worse prognosis (Foulkes et al., 2010; Pandya-Jones et al., 2020).

Recently, it has been reported that RNA and proteins can interact with each other to form a droplet-like unit by multivalent weak interactions based on intrinsically disordered regions (IDR), folded proteins, DNA/RNA molecular scaffolds, and other structures, which was called a liquid-liquid phase separation (LLPS) (Alberti et al., 2019; Li et al., 2021a). Many biological processes, including transcription, chromatin organization, X chromosome inactivation (XCI), DNA damage response (DDR), autophagy, and even tumor growth and metastasis, have been proven to involve LLPS to achieve their specific functions (Du and Chen, 2018; Hahn, 2018; Ries et al., 2019). For example, the YAP protein formed a liquid aggregate in the nucleus, which induced the transcription of its target genes and promoted the growth of MBA-MD-231 breast cancer cells *in vivo* and in vitro (Li et al., 2021a).

LLPSs appear at different phases and spaces in cells to perform specific functions. The components involved in the formation of LLPS can be quite different. Recent studies have shown that long noncoding RNAs (lncRNAs) can regulate cellular functions by interacting with target proteins to form dynamic LLPS (Pandya-Jones et al., 2020). For example, the lncRNA Xist formed condensates in the inactive X(Xi) group by binding to multiple proteins, such as PTBP1, MATR3, TDP-43, and CELF1, with self-aggregation and heterotypic protein-protein interactions, which provided a new way for gene silencing (Pandya-Jones et al., 2020). LncRNA NEAT1 exhibited phase-separated condensate properties, and was able to bind to NONO/SFPQ with the formation of LLPS *in vitro*. In addition, lncRNAs interacted with oncogenes to form LLPS, which were involved in regulating tumor development (Yamazaki et al., 2018). For example, the lncRNA SNHG9 promoted LATS1 to experience LLPS, which further promoted the YAP signaling pathway-induced growth of breast cancer cells (Li et al., 2021a).

In previous research, immunological genes, autophagy-related genes, and some other genes were reported to be useful in tumor prognosis prediction (Shen et al., 2020; Li et al., 2021b; Jiang et al., 2021; Jiang et al., 2022), but few studies focused on the roles of LLPS-related genes (LCGs) in tumor prognosis prediction. For example, prognosis prediction models were based on previously constructed LCGs for ovarian cancer, lung squamous cell carcinoma, and glioma (Qiu et al., 2021; Zheng et al., 2022); the risk model based on LCGs identified a good/bad prognosis cluster. However, an LCG-based risk model has not been reported for breast cancer and its subtypes; so, in this study we constructed a nomogram based on LCGs.

## Methods and materials

### Data collection and collation

**FIGURE 1:**
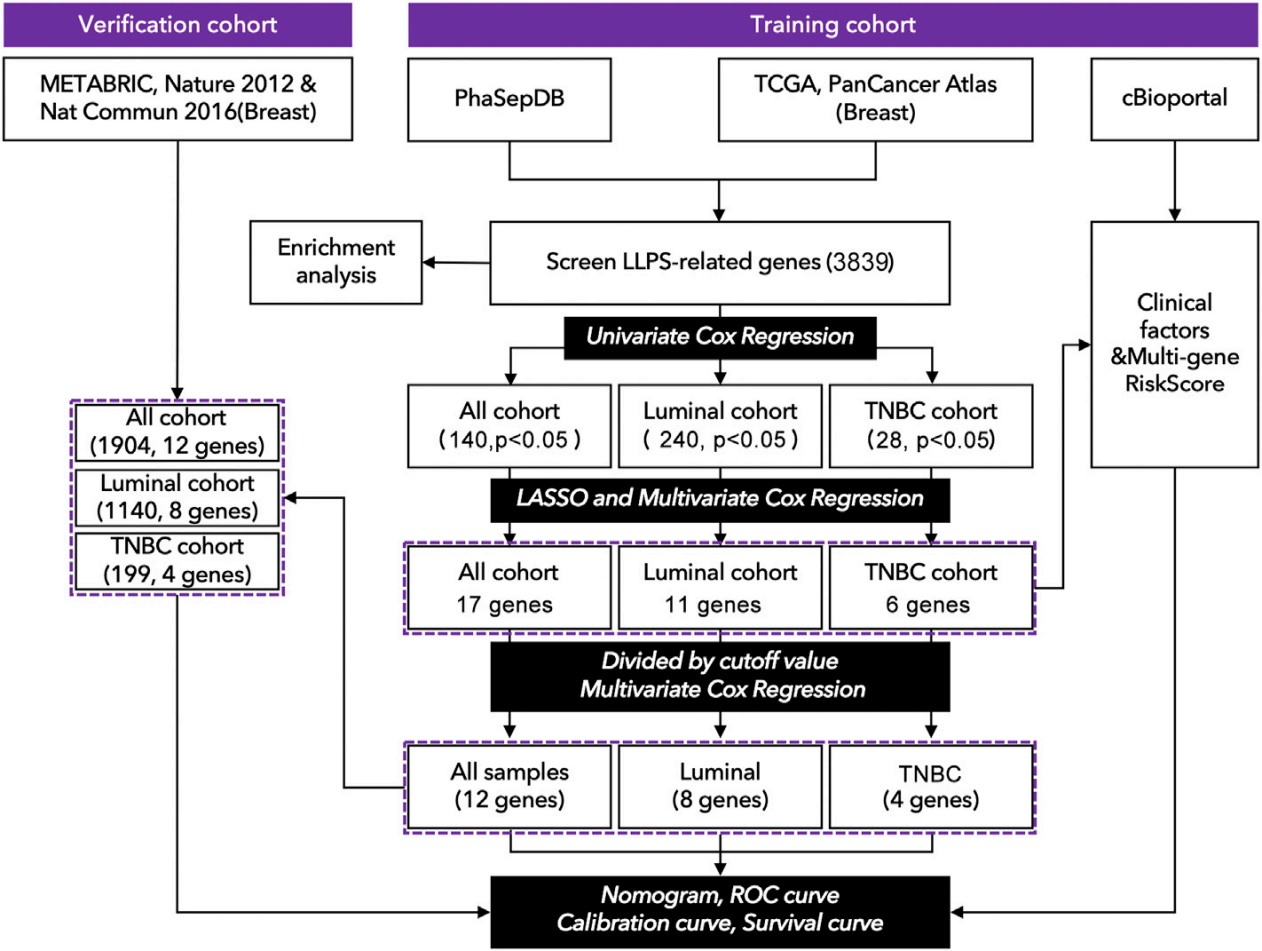
Technology roadmap of study. (1) Gene expression profiles were collected from TCGA: https://portal.gdc.cancer.gov. (2) Clinical characteristics were collected from cBioportal http://www.cbioportal.org. (3) The genomic list of LLPS-related genes was collected from PhaSepDB: http://db.phasep.pro. (4) Data analysis was performed in R4.0.1 and SPSS 20.0.

Gene expression profiles of the training cohort were collected from The Cancer Genome Atlas (TCGA, https://portal.gdc.cancer.gov), and the data of the verification cohort was collected from cBioportal (METRABRIC, Nature 2012 & Nat Commun 2016; http://www.cbioportal.org) and GEO58812. The clinical characteristics of TCGA were obtained from cBioportal (http://www.cbioportal.org). LLPS-related genes were selected from PhaSepDB, an online database that records all LLPS-related genes (http://db.phasep.pro). A total of 1077 records (673 in the luminal cohort, 171 in the TNBC cohort, 0 excluded) were selected from the training cohort, and 1904 records were selected (1140 in the luminal cohort, 199 in the TNBC cohort, and 604 were excluded) from the verification cohort (Figure 1). Clinical factors included “Age (<45, 45 ∼ 64, >64)”, “clinical stage (I-II, III-IV)”, “T stage (T1-2, T3-4)”, “N stage (N0, N1-3), M stage, recurrence status, and radiation therapy”, genomic factors included “Tumor mutation burden (TMB)” and “risk-score (multi-gene risk-score)”.

### Identification of prognostic signature LCGs and construction of an LCG-based risk-score

The gene expression profile was collected from TCGA, and the LCGs were exported from PhaSepDB. Next, 3839 genes were identified by taking the intersection between the data from TCGA and PhaSepDB. Univariate Cox regression (*limma* package in R, *p*< 0.01 in all breast and luminal cohorts, *p*< 0.05 in the TNBC cohort, fold changes >1.5) was performed to identify prognosis-related LCGs. Least absolute shrinkage and selection operator (LASSO) Cox regression (*glmnet* package in R) was performed to narrow the array of candidate genes. Multivariate Cox regression was performed in R to select genes for constructing nomograms or multi-gene risk-scores, in which an LLPS-related risk-score was constructed according to the formula:

Riskscore=Exp (gene1)×Coef(gene1)+Exp (gene2)×Coef(gene2)+…

The “*Exp*” mean expression value of a gene in multivariate Cox regression. “*Coef*” mean coefficient of the corresponding gene. Cohorts were divided into “High-risk group” and “Low-risk group” by risk-score.

### Enrichment analysis

A total of 340 differentially-expressed LCGs were identified by KEGG and GO (“*clusterProfiler, org.Hs.eg.db, and enrichplot*” packages in R) analysis.

### Identification of clinical factors and construction of prognosis prediction model

Clinical factors (clinical stage, N stage, T stage, age) and genomic factors (risk-score, TMB) were subjected to univariate Cox regression and multivariate Cox regression analysis in SPSS 20.0. Nomograms, calibration analysis, and Kaplan Meier (K-M) curves were constructed in R (“*survival, rms, regplot, and survminer*” packages), and the C-index was calculated in R (“*survcomp*”).

### Statistics analysis

All data analyses were performed using R4.0.1 and SPSS 20.0 and some data analyses were performed with online tools (http://www.sangerbox.com/tool). All core R codes related to this study were uploaded on ZENODO (https://zenodo.org) [DIO: 10.5281/zenodo.6497469; link: https://doi.org/10.5281/zenodo.6497469]**.**

## Results

**Identification of prognosis-related LCGs in subtypes of breast cancer**

As shown in Figure 1, 3839 LCGs were identified by searching the intersection between the data from TCGA and PhaSepDB, and those genes were subjected to univariate Cox regression analysis. The samples of breast cancer from TCGA were divided into all cohorts, the luminal cohort, and the TNBC cohort, in which 140 prognosis-related LCGs (pLCGs) were identified in all cohort (*p* < 0.05, Figure 1), 240 pLCGs were identified in the luminal cohort (*p* < 0.05, Figure 1), and 28 pLCGs were identified in the TNBC cohort (*p* < 0.05, Figure 1).

### Construction of LGCs-based prognosis prediction model

**FIGURE 2:**
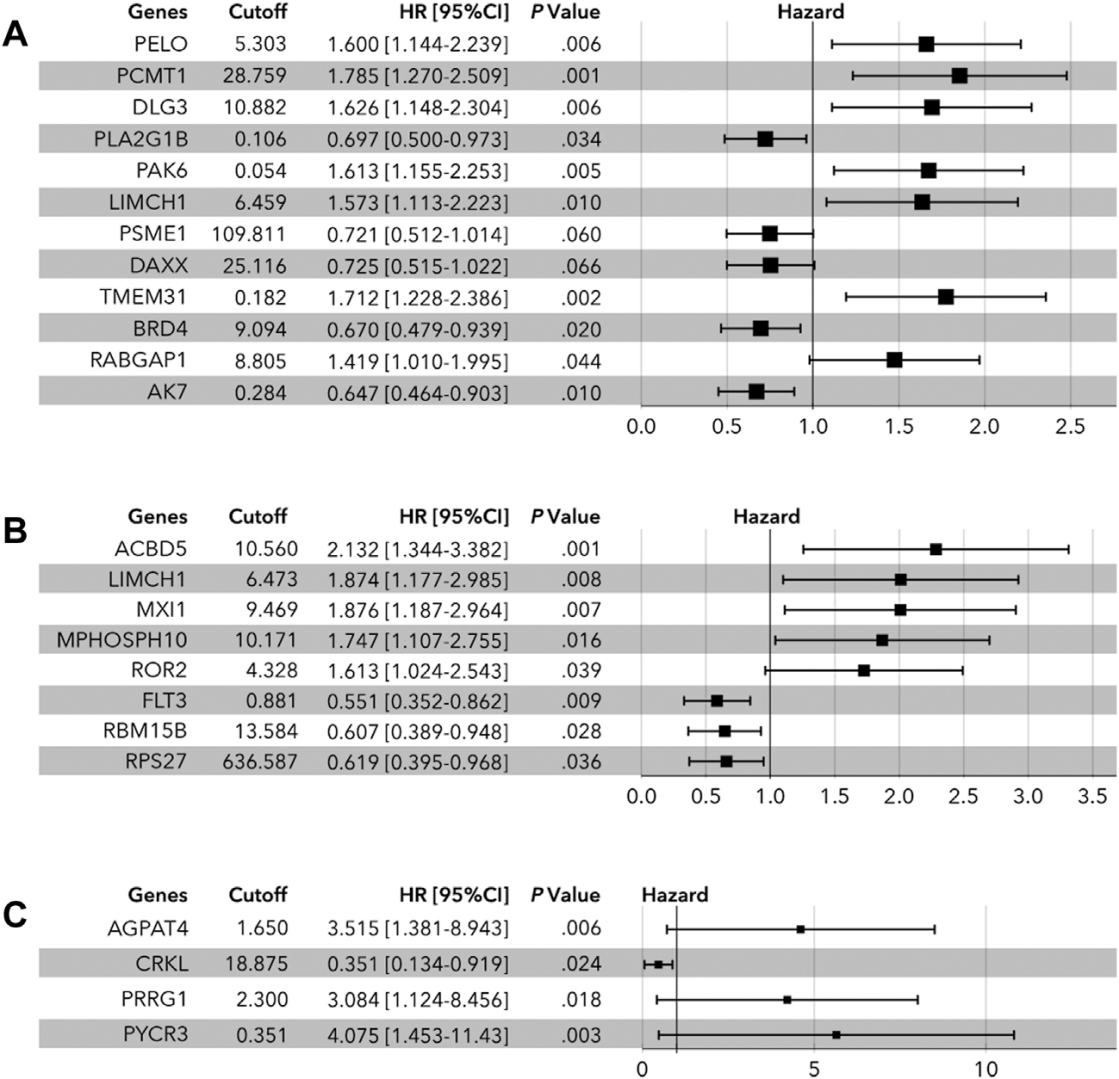
Multivariate Cox regression analysis of LLPS-related genes. **(A)** In all cohort, PELO, PCMT1, DLG3, PLA2G1B, PAK6, LIMCH1, PSME1, DAXX, TMEM31, BRD4, RABGAP1, and AK7 were identified to determine the risk-score. **(B)** In the luminal cohort, ACBD5, LIMCH1, MXI1, MPHOSPH10, ROR2, FLT3, RBM15B, and ROS27 were identified to determine the risk-score. **(C)** In the TNBC cohort, AGPAT4, CRKL, PRRG1, and PYCR3 were identified to calculate the risk-score.

The above candidate genes were subjected to LASSO analysis, and 17 genes in all cohorts were selected for multivariate Cox regression analysis-11 genes from the luminal cohort and 6 genes from the TNBC cohort (Figure 1 and Supplementary Figure S1). The samples were divided into high-expression and low-expression groups by median expression of the selected genes. Then, by multivariate Cox regression analysis, we identified **PELO** (1.600[1.44–2.239], *p* = 0.006); **PCMT1** (1.785[1.270–2.509], *p* = 0.001); **DLG3** (1.626[1.148–2.304], *p* = 0.006); **PLA2G1B** (0.697[0.500–0.973], *p* = 0.034); **PAK6** (1.613[1.155–2.253], *p* = 0.005); **LIMCH1** (1.573[1.113–2.223], *p* = 0.010); **PSME1** (0.721[0.512–1.014], *p* = 0.060); **DAXX** (0.725[0.515–1.022], *p* = 0.066); **TMEM31** (1.712[1.228–2.386], *p* = 0.002); **BRD4** (0.670[0.479–0.939], *p* = 0.020); **RABGAP1** (1.419[1.010–1.995], *p* = 0.044); and **AK7** (0.647[0.464–0.903], *p* = 0.010) as factors for constructing prognosis prediction model in all cohorts (Figure 2A). We also identified 11 genes: **ACBD5** (2.132[1.344–3.382], *p* = 0.001); **LIMCH1** (1.874[1.177–2.985], *p* = 0.008); **MXI1** (1.876[1.187–2.964], *p* = 0.007); **MPHOSPH10** (1.747[1.107–2.755], *p* = 0.016); **ROR2** (1.613[1.024–2.543], *p* = 0.039); **FLT3** (0.551[0.352–0.862], *p* = 0.009); **RBM15B** (0.607[0.389–0.948], *p* = 0.028); and **RPS27** (0.619[0.395–0.968], *p* = 0.036) as factors for constructing a prognosis prediction model in the luminal cohort (Figure 2B). We also identified the six genes: **AGPAT4** (3.515[1.381–8.943], *p* = 0.006); **CRKL** (0.351[0.134–0.919], *p* = 0.024); **PRRG1** (3.084[1.124–8.456], *p* = 0.018); and **PYCR3** (4.075[1.453–11.430], *p* = 0.003) as factors for constructing a prognosis prediction model in the TNBC cohort (Figure 2C).

**FIGURE 3:**
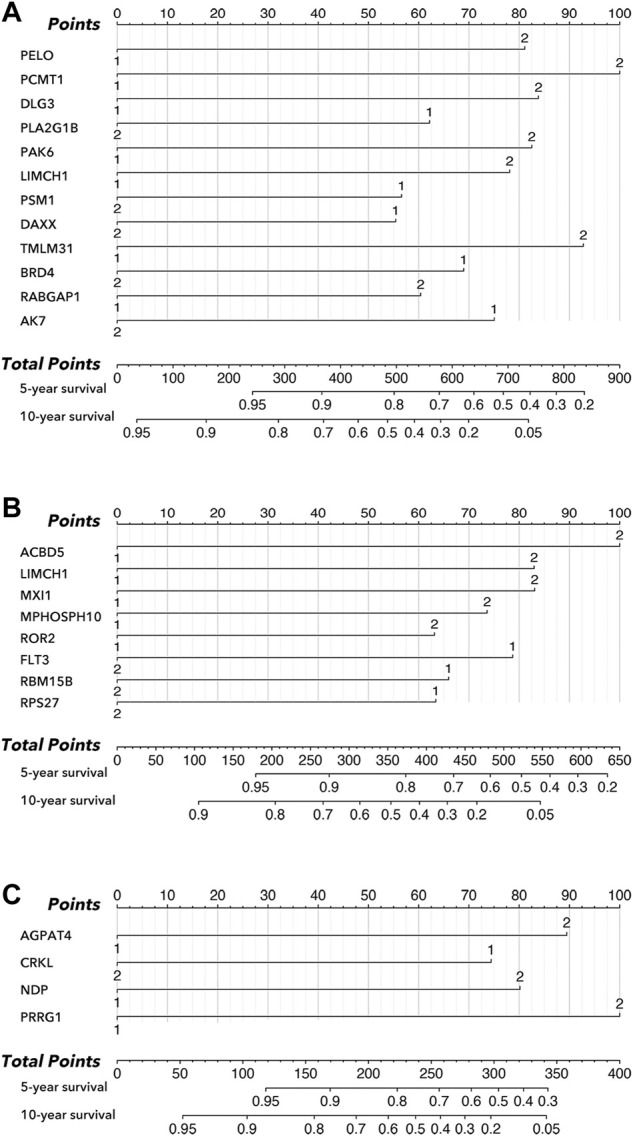
Nomogram in subtypes of breast cancer. **(A)** Nomogram of all cohort, amongst which ‘1’ mean low expression, and ‘2’ mean high expression. **(B)** Nomogram of the luminal cohort, amongst which ‘1’ mean low expression, and ‘2’ mean high expression. **(C)** Nomogram of TNBC cohort, amongst which ‘1’ mean low expression, and ‘2’ mean high expression.

**FIGURE 4:**
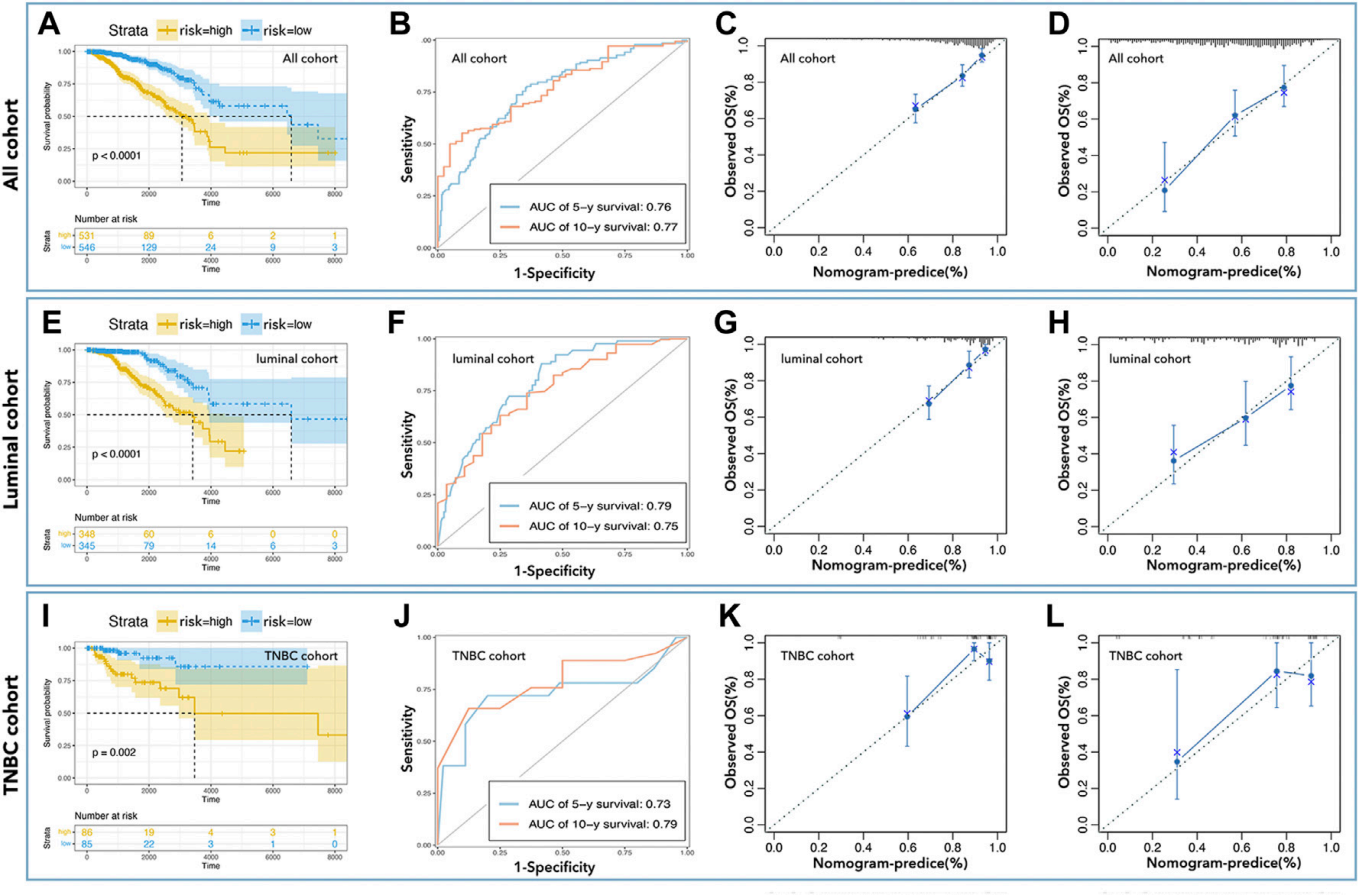
Inner verification of monogram. K-M curve showed low-risk with better prognosis in all cohorts **(A)**, the luminal cohort **(E)**, and the TNBC cohort **(I)**. The AUC values of nomograms in all cohorts **(B)** were 0.76 (5-year survival) and 0.77 (10-year survival), in the luminal cohort **(F)** were 0.79 (5-year survival) and 0.75 (10-year survival), and in all cohort **(J)** were 0.73 (5-year survival) and 0.79 (10-year survival). Calibration analysis was performed to assess the prediction accuracy of nomograms in all cohorta **(C,D)**, luminal cohort **(G,H)**, and TNBC cohort **(K,L)**.

As shown in Figure 3, an LCG-based nomogram was constructed for the training cohort (all cohort, luminal cohort, and TNBC cohort), in which ‘1’ mean low expression and ‘2’ mean high expression. Samples were divided into low-risk and high-risk groups by predicted risk-score calculated by multivariate Cox regression analysis. As shown in Figure 4, the low-risk group had a better prognosis than the high-risk group in all cohorts (Figure 4A, *p* < 0.0001), the luminal cohort (Figure 4E, *p* < 0.0001), and the TNBC cohort (Figure 4I, *p* = 0.002). The ROC value of the nomograms in all cohorts was 0.76 (5-year survival) and 0.77 (10-year survival) (Figure 4B); in the luminal cohort it was 0.79 (5-year survival) and 0.75 (10-year survival) (Figure 4F); and in all cohorts it was 0.73 (5-year survival) and 0.79 (10-year survival) (Figure 4J). In addition, calibration analysis was performed to assess the predictive ability of the nomogram (Figures 4C,D,G,H,K,L).

**FIGURE 5:**
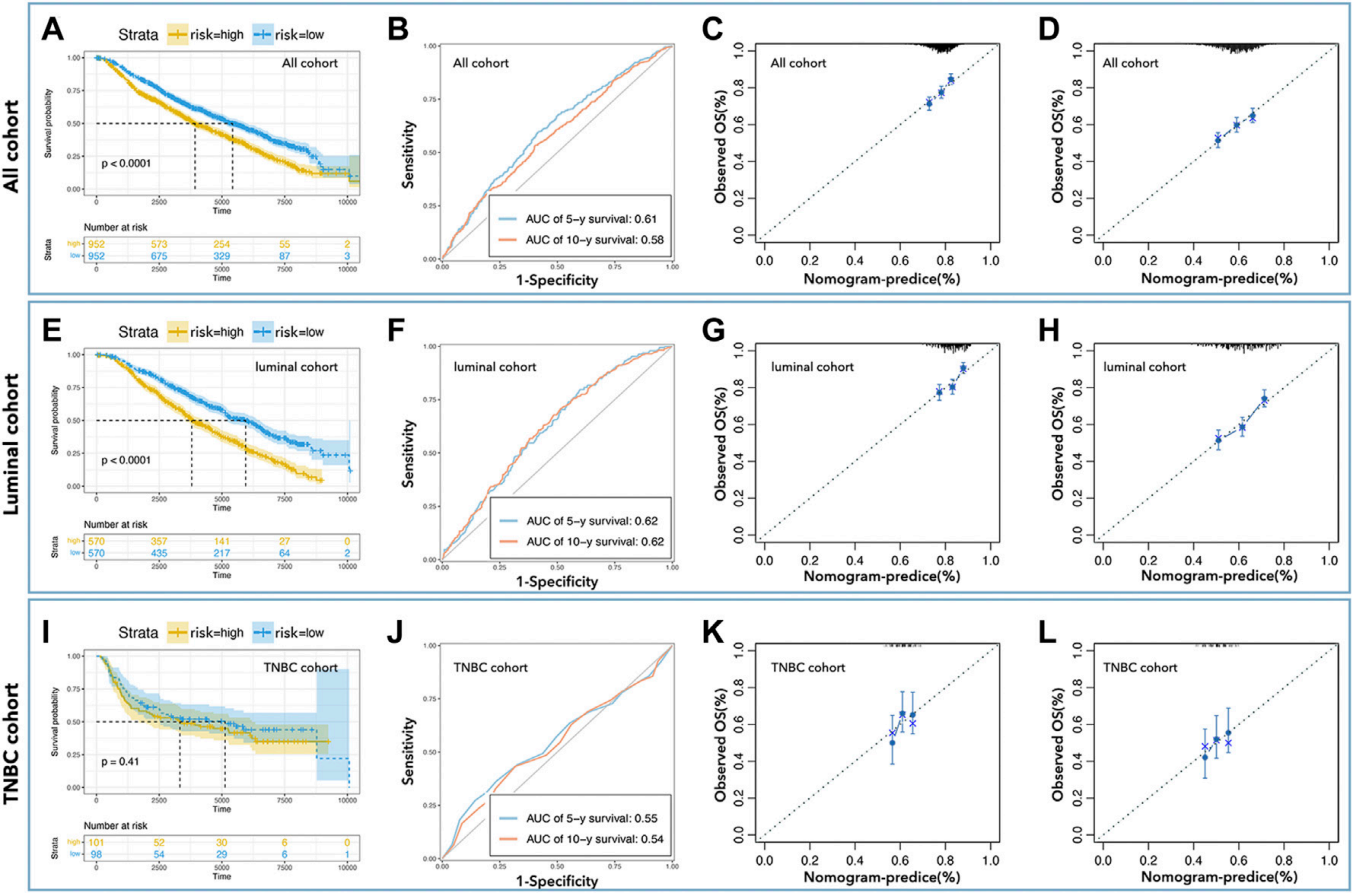
External verification of predicted models. K-M curve showed low-risk with better prognosis in all cohorts **(A)** and the luminal cohort **(E)**, but not in the TNBC cohort **(I)**. The AUC values of nomogram in all cohorts **(B)** were 0.61 (5-year survival) and 0.58 (10-year survival), in the luminal cohort **(F)** were 0.62 (5-year survival, 10-year survival), and in the TNBC cohort **(J)** were 0.55 (5-year survival) and 0.54 (10-year survival). Calibration analysis was performed to assess the prediction accuracy of nomograms in all cohorts **(C,D)**, the luminal cohort **(G,H)**, and the TNBC cohort **(K,L)**.

To further verify the prediction ability of the above nomogram (prognosis prediction model), data from METABRIC (Nature 2012 & Nat Commun 2016) was used to construct the verification cohort (Figure 1). We used the above-identified genes to construct the prediction model in all cohorts, the luminal cohort, and the TNBC cohort. As shown in Figure 5, the high-risk group had a worse prognosis than the low-risk group in all cohorts (Figure 5A, *p*< 0.0001) and the luminal cohort (Figure 5E, *p*< 0.0001), while there was no difference in the TNBC cohort (Figure 5I, *p* = 0.41). In addition, the ROC values of the nomogram in the verification cohort were not good: 0.61 (5 years) and 0.58 (10 years) in all cohorts (Figure 5B), 0.62 (5 years, 10 years) in the luminal cohort (Figure 5F), and 0.56 (5 years) and 0.54 (10 years) in the TNBC cohort (Figure 5J). Calibration analysis was also performed (Figures 5C,D,G,H,K,L).

### Enrichment analysis

**FIGURE 6:**
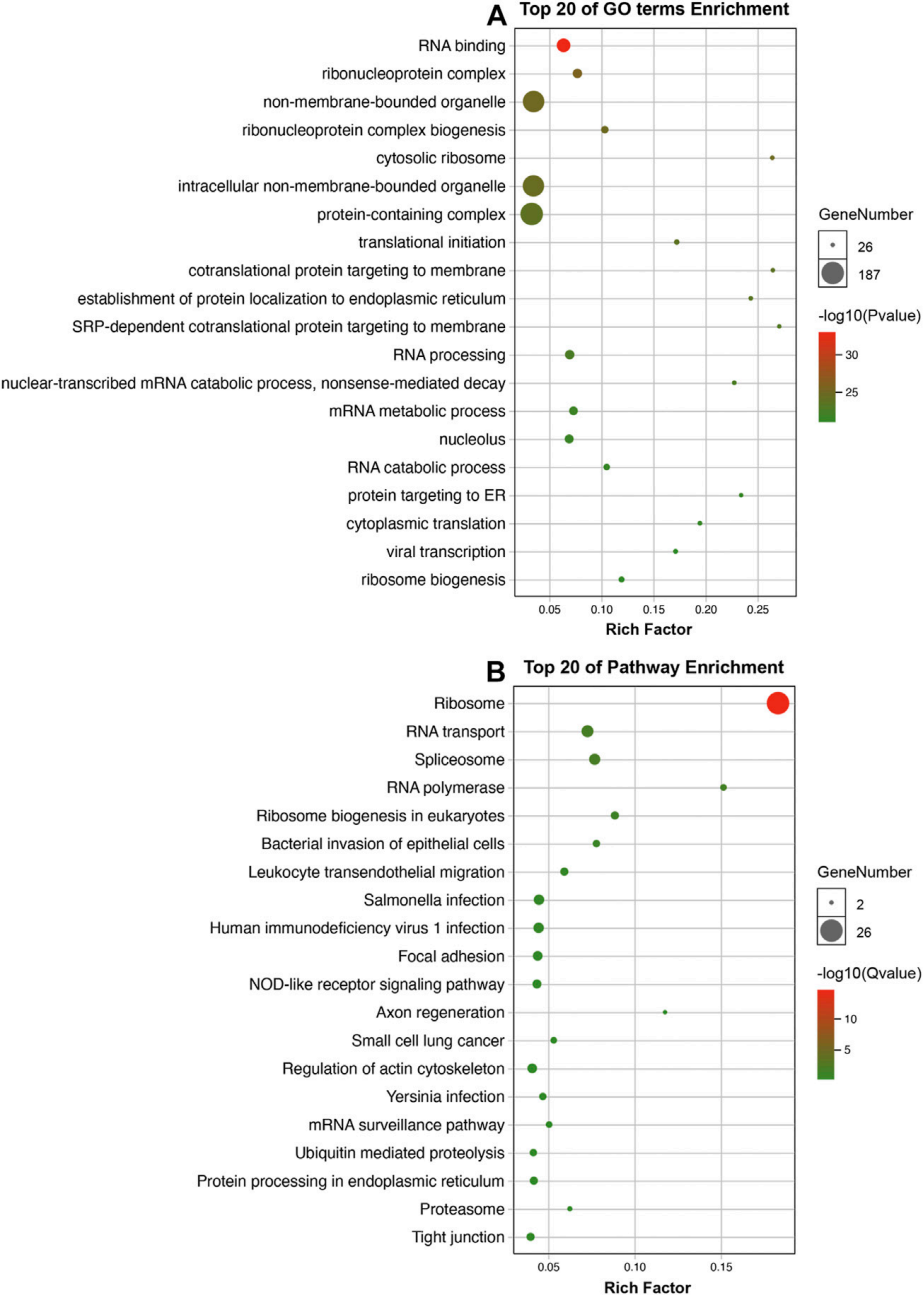
Enrichment analysis. **(A)** GO analysis of LLPS-related genes; **(B)** KEGG analysis of LLPS-related genes.

A total of 340 differentially-expressed LCGs were subjected to KEGG and GO analysis to identify molecular signaling pathways. As the GO analysis showed, LCGs were involved in RNA binding, protein targeting to ER, and translational initiation, etc. (Figure 6A). KEGG analysis showed that LCGs were involved in the NOD-like receptor signaling pathway, focal adhesion, tight junctions, and spliceosomes, etc. (Figure 6B).

### Prognosis prediction model based on LCGs risk-score

**FIGURE 7:**
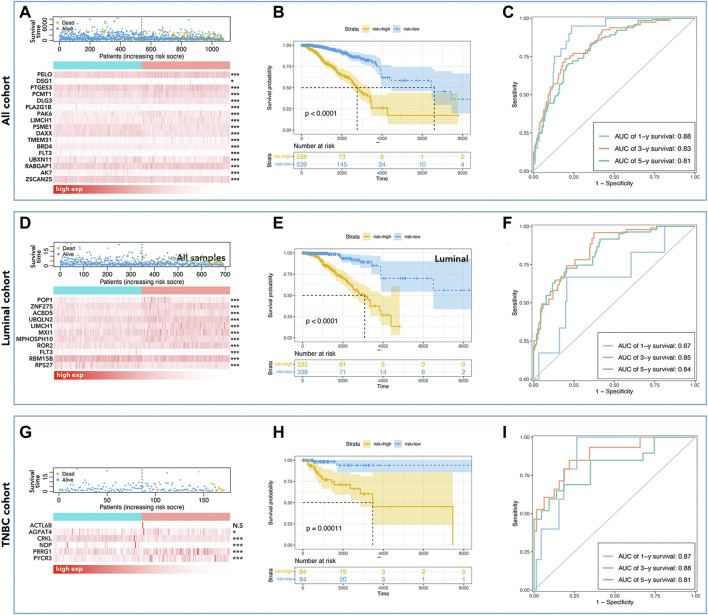
LLPS-related multi-gene risk-score. Risk-score-mortality relationship and selected LLPS-related gene expression differences are shown for all cohort **(A)**, luminal cohort **(D)**, and TNBC cohort **(G)**. The K-M curve showed that low-risk had better prognosis in all cohorts **(B)**, the luminal cohort **(E)**, and in the TNBC cohort **(H)**. The AUC values of risk-score in all cohorts **(C)** were 0.88 (1 year), 0.83 (3 years), and 0.81 (5 years), in the luminal cohort **(F)** were 0.67 (1 year), 0.85 (3 years), and 0.84 (5 years), and in the TNBC cohort **(I)** were 0.87 (1 year), 0.88 (3 years), and 0.81 (5 years).

To further explore the effects of LCGs on the prediction of prognosis, multi-gene risk-scores were calculated. As shown in Figure 7, 17 pLCGs were used to obtain multi-gene risk-scores in all cohorts, all of which had different expressions between tumor and non-tumor tissues (Figure 7A); 11 pLCGs were used to determine risk-scores in the luminal cohort, all of which had different expressions between tumor and non-tumor tissues (Figure 7D); 6 pLCGs were used to calculate risk-scores in the TNBC cohort, and *AGPAT4*, *CRKL*, *NDP*, *PRRG1*, and *PYCR3* all had different expression between tumor and non-tumor tissues (Figure 7G). The K-M curve showed that the group with the lowest risk-score had the best prognosis (**Fig. B, E, and H**). We determined the prognostic prediction ability of risk-scores in breast cancer and its subtypes, and found that the ROC value of the risk-score based on 17 LCGs was 0.88 (1 year), 0.83 (3 years), and 0.81 (5 years) in all cohorts (Figure 7C); the ROC value of the risk-score based on 11 LCGs was 0.67 (1 year), 0.85 (3 years), and 0.84 (5 years) in the luminal cohort (Figure 7F); the ROC value of the risk-score based on 6 LCGs was 0.87 (1 year), 0.88 (3years), and 0.81 (5 years) in the TNBC cohort (Figure 7I).

### LCG-based risk-score in the regulation of genomic instability and tumor immunity

**FIGURE 8:**
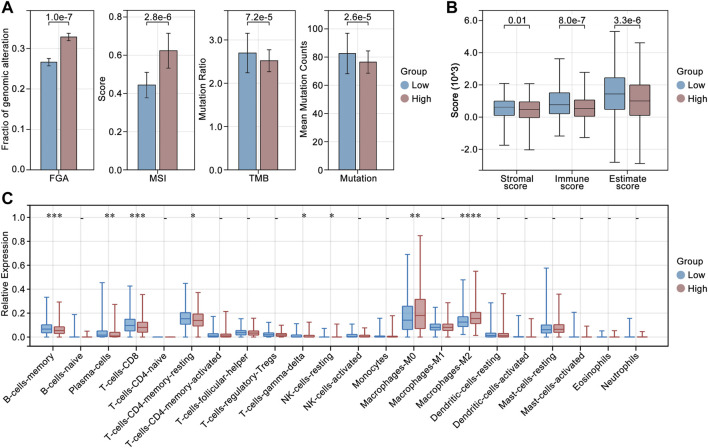
Genomic instability and tumor immunity analysis. **(A)** Differences in fraction of genomic alteration (FGA), microsatellite instability (MSI), gene mutation (mutation) and tumor mutation burden (TMB) in high risk-score and low risk-score groups. **(B)** Immune score in high risk-score and low risk-score groups. **(C)** Differences in immune cell infiltration in high risk-score and low risk-score groups.

To explore the roles of LCGs in the regulation of genomic instability and tumor immunity, we calculated the differences in fraction of genomic alteration (FGA)*,* microsatellite instability (MSI), gene mutation (mutation) and tumor mutation burden (TMB). As shown in Figure 8, the group with higher LCG-based risk-scores also had a higher level of FGA and MSI, while it was accompanied by a lower level of mutation and TMB. In immunity analysis, we found that the group with the higher LCG-based risk-score was accompanied by a lower immunity score independent of the StromalScore, ImmuneScore, and EstimateScore (Figure 8B). In addition, we explored the differences in immune cell infiltration. As shown in Figure 8C, a higher LCG-based risk-score group was accompanied by a lower infiltration of memory B cells, plasma cells, CD8^+^ T cells, resting memory CD4^+^ T cells, γδ T cells, and resting NK cells, while it was accompanied by a higher infiltration of M0 macrophages and M2 macrophages (Figure 8C).

### Prognosis prediction model based on LCG-based risk-score and clinical factors

**FIGURE 9:**
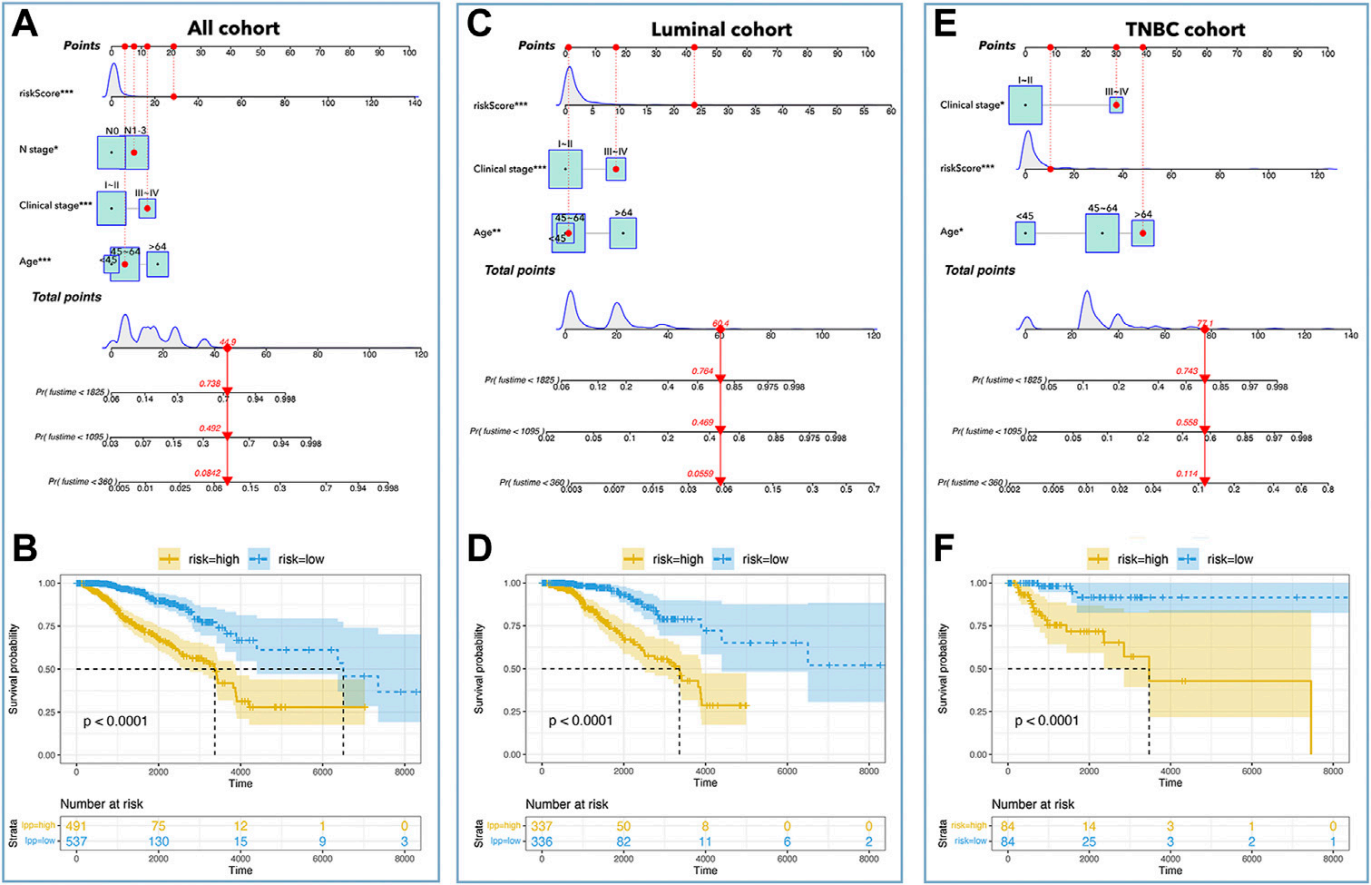
Nomograms in subtypes of breast cancer. Nomograms of all cohorts **(A)**, luminal cohort **(C)**, and TNBC cohort **(E)**. The K-M curve showed that a low-risk-score had better prognosis in all cohorts **(B)**, luminal cohort **(D)**, and TNBC cohort **(F)**.

For further development of the prognosis prediction model, we put clinical data into the construction of nomograms. Table 1 showed the clinical characteristics and genomic features of breast cancer (TCGA), and we excluded ‘recurrence, M stage, radiation’ whose proportion of subgroups was <10%. We identified ‘age, clinical stage, N stage, and risk-score’ as factors for constructing nomograms by univariate Cox regression and multivariate Cox regression in all cohorts (Table 2); we identified ‘age, clinical stage, and risk-score’ as factors for constructing nomograms in the luminal cohort and the TNBC cohort (Table 2). As shown in Figure 9, the total points provided a point-to-point survival prediction, such that a score of 44.9 corresponded to a 5-year death probability of 73.8%, a 3-year death probability of 49.2%, and a 1-year death probability of 8.42% in all cohort breast samples (Figure 9A). The same methods for prognosis prediction were used in the luminal and TNBC cohorts (Figures 9C,E). We divided the sample nomograms into a low-risk group and a high-risk group and the results showed that the low-risk group displayed a better prognosis than the high-risk group (Figures 9B,D,F, *p* <0.0001).

**TABLE 1:** | Clinical and genomic characteristics of subtypes of breast cancers. (1) Data from TCGA, from which the total sample was 1077 records, the luminal cohort was 693 records, the TNBC cohort was 171 records, and the other cohort was 213 records. (2) The asterisk * means that factors whose proportion of subtypes was <10% were selected for further analysis.

Item		All samples (*n* = 1077)	Luminal (*n* = 693)	TNBC (*n* = 171)	Others (*n* = 213)
Clinical characteristics		Number (%)			
*Age (years)* *^a^*	*1 (<45)*	158 (14.7)	98 (14.1)	32 (18.7)	28 (13.1)
	*2 (45∼64)*	584 (54.2)	360 (51.9)	97 (56.7)	127 (59.6)
	*3 (>64)*	335 (31.1)	235 (33.9)	42 (24.6)	58 (27.2)
*Recurrence*	*1 (Yes)*	100 (9.3)	58 (10.4)	16 (9.4)	26 (12.2)
	*2 (No)*	778 (72.2)	501 (72.3)	125 (73.1)	152 (71.4)
	*3 (Na)*	199 (18.5)	134 (19.3)	30 (17.5)	35 (16.4)
*M stage*	*1 (No)*	896 (83.2)	575 (83.0)	149 (87.1)	172 (80.8)
	*2 (Yes)*	21 (1.9)	12 (1.7)	3 (1.8)	6 (2.8)
	*3 (Na)*	160 (14.9)	106 (15.3)	19 (11.1)	35 (16.4)
*N stage* *^a^*	*1 (N0)*	510 (47.4)	314 (45.3)	107 (62.6)	89 (41.8)
	*2 (N1-3)*	547 (50.8)	365 (52.7)	64 (37.4)	118 (55.4)
	*3 (Na)*	20 (1.9)	14 (2.0)	0 (0.0)	6 (2.8)
*T stage* *^a^*	*1 (T1-2)*	899 (83.5)	584 (84.3)	149 (87.1)	166 (77.9)
	*2 (T3-4)*	174 (16.2)	106 (15.3)	21 (12.3)	47 (22.1)
	*3 (Na)*	4 (0.4)	3 (0.4)	1 (0.6)	0 (0.0)
*Clinical stage* *^a^*	*1 (I∼II)*	793 (73.6)	505 (72.9)	145 (84.8)	143 (67.1)
	*2 (III∼IV)*	266 (24.7)	176 (25.4)	23 (13.5)	67 (31.5)
	*3 (Na)*	18 (1.7)	12 (1.7)	3 (1.8)	3 (1.4)
*Radiation*	*1 (yes)*	977 (90.7)	634 (91.5)	154 (90.1)	189 (88.7)
	*2 (No)*	0 (0.0)	0 (0.0)	0 (0.0)	0 (0.0)
	*3 (Na)*	100 (9.3)	59 (8.5)	17 (9.9)	24 (11.3)
Genomic characteristics		Median ± Sd.			
*TMB* *^a^*		2.746 ± 9.317	2.546 ± 9.729	3.350 ± 4.815	2.924 ± 10.723

TMB: tumor mutation burden

a Factors are selected into further analysis whose proportion of subgroups ≥ 10%.

**TABLE 2:** | Identifying clinical factors in the nomogram. (1) Age, clinical stage, N stage, and risk-score were selected in all cohorts; (2) Age, clinical stage, and risk-score were selected in the luminal cohort; (3) Age, clinical stage, and risk-score were selected in the TNBC cohort.

Item			Univariate cox Analysis			Multivariate cox Analysis		
			HR	95% Cl	*p* value	HR	95% Cl	*p* value
All	Age*	<45						
		45∼64	1.078	0.650–1.788	0.771	1.359	0.813–2.269	0.242
		>64	2.354	1.406–3.943	0.001	2.884	1.702–4.886	<0.001
	TMB		0.967	0.898–1.042	0.380			
	Clinical stage*	I∼II					–	
		III∼IV	2.789	1.982–3.923	<0.001	2.275	1.489–3.476	<0.001
	T stage	T1∼2						
		T3∼4	1.871	1.278–2.741	0.001			
	N stage*	N0						
		N1∼3	2.215	1.543–3.181	<0.001	1.679	1.067–2.643	0.025
	RiskScore*		1.051	1.039–1.063	<0.001	1.051	1.039–1.063	<0.001
TNBC	Age*	<45						
		45∼64	0.831	0.273–2.529	0.745	3.460	0.657–18.226	0.143
		>64	1.930	0.590–6.320	0.277	6.664	1.272–34.922	0.025
	TMB		0.830	0.597–1.153	0.267			
	Clinical stage*	I∼II						
		III∼IV	6.284	2.555–15.454	<0.001	4.340	1.397–13.486	0.011
	T stage	T1∼2						
		T3∼4	2.429	0.802–7.356	0.116			
	N stage	N0						
		N1∼3	4.123	1.596–10.649	0.003			
	RiskScore*		1.038	1.025–1.051	<0.001	1.040	1.021–1.059	<0.001
Luminal	Age*	<45						
		45∼64	0.886	0.445–1.765	0.732	1.054	0.520–2.135	0.885
		>64	2.276	1.159–4.470	0.017	2.639	1.308–5.232	0.007
	TMB		1.006	0.959–1.055	0.804			
	Clinical stage*	I∼II						
		III∼IV	2.289	1.467–3.571	<0.001	2.340	1.471–3.720	<0.001
	T stage	T1∼2						
		T3∼4	1.643	0.995–2.714	0.053			
	N stage	N0						
		N1∼3	1.881	1.183–2.991	0.008			
	RiskScore*		1.104	1.081–1.128	<0.001	1.096	1.071–1.121	<0.001

**FIGURE 10:**
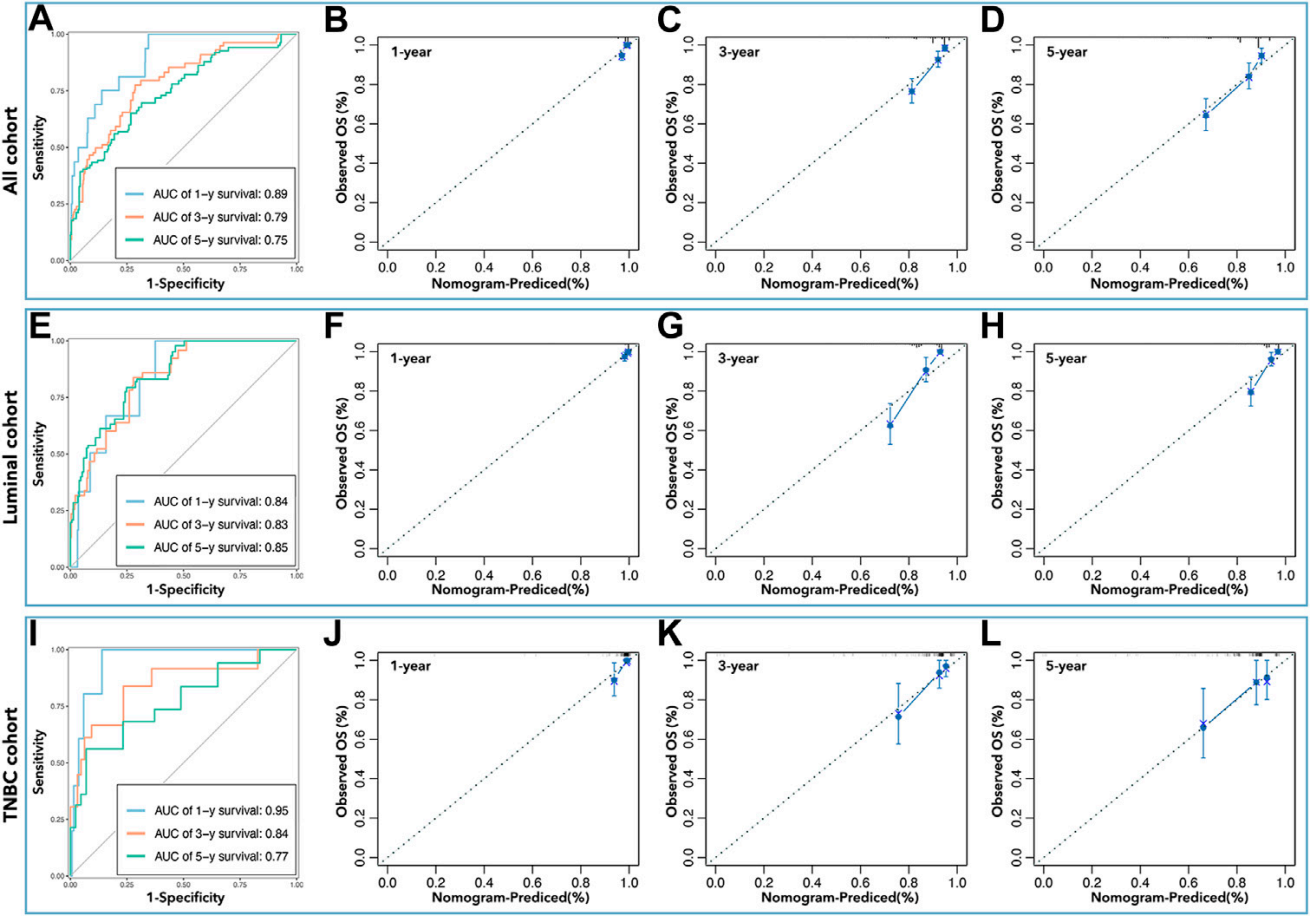
Inner verification of nomograms. The AUC values of nomograms in all cohorts **(A)** were 0.89 (1 year), 0.79 (3 years), and 0.75 (5 years), in the luminal cohort **(E)** were 0.84 (1 year), 0.83 (3 years), and 0.85 (5 years), and in the TNBC cohort **(I)** were 0.95 (1 year), 0.84 (3 years), and 0.77 (5 years). Calibration analysis was performed to assess the prediction accuracy of nomograms in all cohorts **(B–D)**, the luminal cohort **(F–H)**, and the TNBC cohort **(J–L)**.

Inner verification was performed to assess the predictive ability of the above nomograms. ROC curves showed that the AUC values were 0.89 (1-year survival), 0.79 (3 years), and 0.75 (5 years) in all cohorts (Figure 10A); the AUC values were 0.84 (1-year survival), 0.83 (3 years), and 0.85 (5 years) in the luminal cohort (Figure 10E); and the AUC values were 0.95 (1-year survival), 0.84 (3 years), and 0.77 (5 years) in the TNBC cohort (Figure 10I). Calibration analysis was shown in Figure 10, which implied that nomograms were accurate in their prediction of breast cancer prognosis and its subtypes. The C-index was also calculated to assess the predictive ability of the nomograms, and Table 3 shows that the C-index was 0.784 [0.741–0.827] for nomograms in all cohorts, 0.803 [0.756–0.850] for nomograms in the luminal cohort, and 0.847 [0.759–0.934] for nomograms in the TNBC cohort.

**TABLE 3:** | C-index of nomograms. The C-index value of nomograms was 0.784 [0.741–0.827] in all cohorts, 0.803 [0.756–0.850] in the luminal cohort, and 0.847 [0.759–0.934] in the TNBC cohort.

Item	C index	Lower value	Upper value
All cohort	0.784	0.741	0.827
Luminal cohort	0.803	0.756	0.850
TNBC cohort	0.847	0.759	0.934

## Discussion

According to the National Cancer Report 2019, breast cancer has become the most common type of tumor in women, with more than 300,000 new breast cancers and more than 66,000 deaths every year (Siegel et al., 2020). Amongst them, HER2-negative breast cancer has to rely on paclitaxel-based combination chemotherapy because of the lack of effective molecular targeted therapy strategies. However, continuous low-sensitivity chemotherapy can easily cause drug resistance, reduce chemotherapy’s clinical benefits, and increase the risk of recurrence and metastasis (Foulkes et al., 2010). Therefore, the development of sensitive chemotherapy is pivotal to the current clinical treatment of breast cancer, but is still a very difficult problem in scientific research.

In eukaryotic cells, there are many structures lacking membranes such as nucleoli, premature cell leukemia nuclei (PML NB), P bodies in *C. elegans*, etc., and they perform key functions in metabolic processes (Boeynaems et al., 2018). Previous studies have pointed out that membrane-free structures are formed from LLPS, which is also named membrane-free condensate or biological condensate. Recently, it was reported that LLPS was involved in neurological diseases and tumor processes. For example, tau species that formed LLPS under cellular conditions could serve as intermediates for tau aggregate formation (Wegmann et al., 2018); cAMP-dependent protein kinase (type I regulatory subunit) produced LLPS as part of their functional role in cAMP signaling to form biomolecular condensates enriched in cAMP and PKA activity, which was critical for effective cAMP compartmentation and played roles in atypical liver cancer (Zhang et al., 2020); YAP protein formed a liquid aggregate in the nucleus and promoted the growth of breast cancer cells by inducing the transcription of oncogenes (Li et al., 2021a). In addition, some studies were reported to apply LCGs to construct multi-gene risk-scores to assess the effects of LLPS in the prediction of tumor prognosis in ovarian epithelial cancer and lung cancer (Qiu et al., 2021; Zhuge et al., 2021). These two studies showed that LCGs were useful in distinguishing subgroups in which one group had a better prognosis, while the other had a worse prognosis. However, the visual prognosis prediction tool used was not given. So, we intended to use LCGs and clinical factors to construct visual prognosis prediction tools.

In this study, we identified differentially expressed pLCGs in breast cancer, and then selected pLCGs as factors to construct the prognosis prediction tool. Twelve genes were identified and samples were divided into low-expression and high-expression groups by the expression of those 12 genes in all cohorts. Although the 12-gene-based nomogram showed medium accuracy in the prediction of prognosis (AUC>0.7, Figure 4A), its performance in inner verification was not good enough (0.7 > AUC>0.5, Figure 5B) to apply it in the prediction of breast cancer prognosis. It was worse in the prediction of 1-year survival and 3-year survival in breast cancer (all cohorts) (data not shown). Meanwhile, we observed that the results in the luminal and TNBC cohorts were too weak to be useful. However, to our surprise, the LCG-based risk-score exactly divided breast cancer samples into better prognosis groups and worse prognosis in all cohorts, the luminal cohort, and the TNBC cohorts (Figures 7B,E,H). Afterwards, we applied LCG-based risk-scoring to construct a nomogram. Although the ROC curve displayed medium strength in the prediction of prognosis (AUC value > 0.8, Figures 7C,F,I; C-index>0.7, data not shown), calibration analysis showed that the results were not good enough (data not shown). So, we combined risk-score and clinical factors to construct a better nomogram for the prediction of prognosis in breast cancer. Inner verification showed that the LLPS-related-gene-based and clinical-factor-based nomograms gave good results for prediction of breast cancer prognosis, especially in the TNBC cohort, for which the AUC value (Figure 10I) of 1-year survival prediction was 0.95, 3-year survival prediction was 0.84, 5-year survival prediction was 0.77, C-index was 0.847 (Table 3), and the calibration analysis results were good (Figures 10J–L). Unfortunately, we did not find any available data that contained both gene expression profiles and clinical characteristics together to perform external verification. However, we still performed independent verification by GEO data. As shown in Supplementary Figure S2, the LCG-based risk-score results were not good enough. The ROC value of 1/3/5/7-year overall survival were 0.30, 0.58, 0.64 and 0.61 (Supplementary Figure S2A), while the ROC value of 1/3/5/7-year metastasis-free survival were 0.30, 0.58, 0.64 and 0.61 (Supplementary Figure S2B). Furthermore, the LCG-based risk-score and clinical factor (age) were applied simultaneously to construct a nomogram, and the results showed that the ROC values for 1/3/5/7-year overall survival were 0.90, 0.7, 0.77 and 0.75 (Supplementary Figure S2C), while the ROC value for 1/3/5/7-year metastasis-free survival were 0.94, 0.74, 0.76 and 0.74 (Supplementary Figure S2D).

In general, this study demonstrated that the prediction ability of nomograms based only on LLPS-related genes was not good enough to be applied in breast cancer therapy. However, the prognosis prediction tools, based on LCG-based risk-scores and clinical factors, had medium accuracy, which means that LCGs are useful for constructing a prognosis prediction model when combined with clinical factors. Next, we will collect our gene expression profiles and clinical data to make an external verification to further assess the prognosis prediction tools in the TNBC cohort.

The limitations of this work are obvious: 1. This work did not detect the real expression levels of those genes used to calculate risk-scores in fresh frozen breast cancer tissues by RNA-sequence assay. The low expression of those selected genes could result in large fluctuations in the assessment of prognosis; 2. Because of limitations in the collection of clinical data (breast cancer has good prognosis, so it is not easy to make a follow-up over 5 years or more in a limited time) this study did not perform external verification. 3. This study did not make a comparison between our prognostic model and another multi-gene-based model in the prediction of breast cancer prognosis.

## Data Availability

The original contributions presented in the study are included in the article/Supplementary Material. Further inquiries can be directed to the corresponding authors.

## Supplementary material

The Supplementary Material for this article can be found online at: https://www.frontiersin.org/articles/10.3389/fgene.2022.834471/full#supplementary-material

Supplementary Figure S1 LASSO analysis to narrow the candidate genes. Binomial deviance and coefficients in all cohorts **(A,D)**, in the luminal cohort **(B,E)**, and the TNBC cohort **(C,F)**.

Supplementary Figure S2 External verification of LCG-based risk-score and age. **(A)** ROC value of 1/3/5/7-year overall survival in LCG-based risk-score. **(B)** ROC value of 1/3/5/7-year metastasis-free survival in LCG-based risk-score. **(C)** ROC value of 1/3/5/7-year overall survival in LCG-based risk-score and age. **(D)** ROC value of 1/3/5/7-year metastasis-free survival in LCG-based risk-score and age.

## Abbreviations

K-M curve, Kaplan Meier curve; FGA, fraction of genomic alteration; LCGs, LLPS-correlated genes; LLPS, liquid-liquid phase separation; pLCGs, prognosis-related LCGs; TCGA, The Cancer Genome Atlas; TNBC, triple negative breast cancer; TMB, tumor mutation burden.
