# Supplementary material for: Comprehensive analysis of liquid-liquid phase separation-related genes in prediction of breast cancer prognosis
Source: Front Genet. 2022 Sep 28;13:834471. doi: 10.3389/fgene.2022.834471 (PMC9554098; doi:10.3389/fgene.2022.834471)
Supplement: Supplementary file 1 [file Table1.docx]

Table 1: Clinical and genomic characteristics of breast cancer (TCGA)

| Item | | All samples  (n=1077) | Luminal  (n=693) | TNBC  (n=171) | Others  (n=213) |
| --- | --- | --- | --- | --- | --- |
| Clinical characteristics | | Number (%) | | | |
| *Age (years)** | *1 (<45)* | 158 (14.7) | 98 (14.1) | 32 (18.7) | 28 (13.1) |
|  | *2 (45~64)* | 584 (54.2) | 360 (51.9) | 97 (56.7) | 127 (59.6) |
|  | *3 (>64)* | 335 (31.1) | 235 (33.9) | 42 (24.6) | 58 (27.2) |
| *Recurrence* | *1 (Yes)* | 100 (9.3) | 58 (10.4) | 16 (9.4) | 26 (12.2) |
|  | *2 (No)* | 778 (72.2) | 501 (72.3) | 125 (73.1) | 152 (71.4) |
|  | *3 (Na)* | 199 (18.5) | 134 (19.3) | 30 (17.5) | 35 (16.4) |
| *M stage* | *1 (No)* | 896 (83.2) | 575 (83.0) | 149 (87.1) | 172 (80.8) |
|  | *2 (Yes)* | 21 (1.9) | 12 (1.7) | 3 (1.8) | 6 (2.8) |
|  | *3 (Na)* | 160 (14.9) | 106 (15.3) | 19 (11.1) | 35 (16.4) |
| *N stage** | *1 (N0)* | 510 (47.4) | 314 (45.3) | 107 (62.6) | 89 (41.8) |
|  | *2 (N1-3)* | 547 (50.8) | 365 (52.7) | 64 (37.4) | 118 (55.4) |
|  | *3 (Na)* | 20 (1.9) | 14 (2.0) | 0 (0.0) | 6 (2.8) |
| *T stage** | *1 (T1-2)* | 899 (83.5) | 584 (84.3) | 149 (87.1) | 166 (77.9) |
|  | *2 (T3-4)* | 174 (16.2) | 106 (15.3) | 21 (12.3) | 47 (22.1) |
|  | *3 (Na)* | 4 (0.4) | 3 (0.4) | 1 (0.6) | 0 (0.0) |
| *Clinical stage** | *1 (I~II)* | 793 (73.6) | 505 (72.9) | 145 (84.8) | 143 (67.1) |
|  | *2 (III~IV)* | 266 (24.7) | 176 (25.4) | 23 (13.5) | 67 (31.5) |
|  | *3 (Na)* | 18 (1.7) | 12 (1.7) | 3 (1.8) | 3 (1.4) |
| *Radiation* | *1 (yes)* | 977 (90.7) | 634 (91.5) | 154 (90.1) | 189 (88.7) |
|  | *2 (No)* | 0 (0.0) | 0 (0.0) | 0 (0.0) | 0 (0.0) |
|  | *3 (Na)* | 100 (9.3) | 59 (8.5) | 17 (9.9) | 24 (11.3) |
| Genomic characteristics | | Median $\pm$ Sd. | | | |
| *TMB** | | 2.746$\pm$9.317 | 2.546$\pm$9.729 | 3.350$\pm$4.815 | 2.924$\pm$10.723 |

TMB: tumor mutation burden

* Factors are selected into further analysis whose proportion of subgroups $\geq$ 10%.
