# Supplementary figures and images for: Comprehensive analysis of liquid-liquid phase separation-related genes in prediction of breast cancer prognosis
Source: Front Genet. 2022 Sep 28;13:834471. doi: 10.3389/fgene.2022.834471 (PMC9554098; doi:10.3389/fgene.2022.834471)

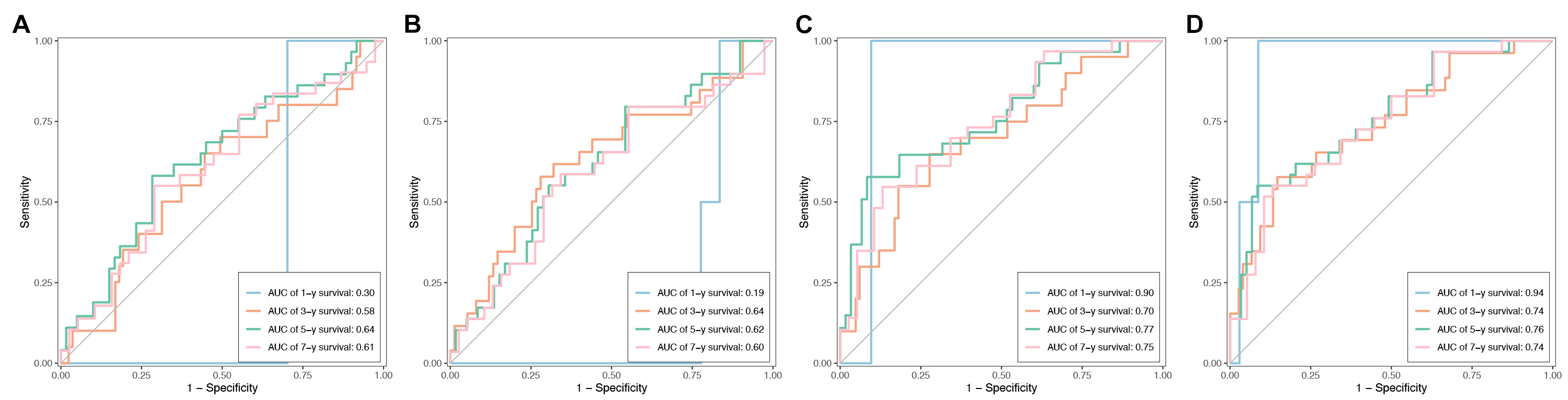

Supplement: Supplementary file 2 [file Image2.tif]

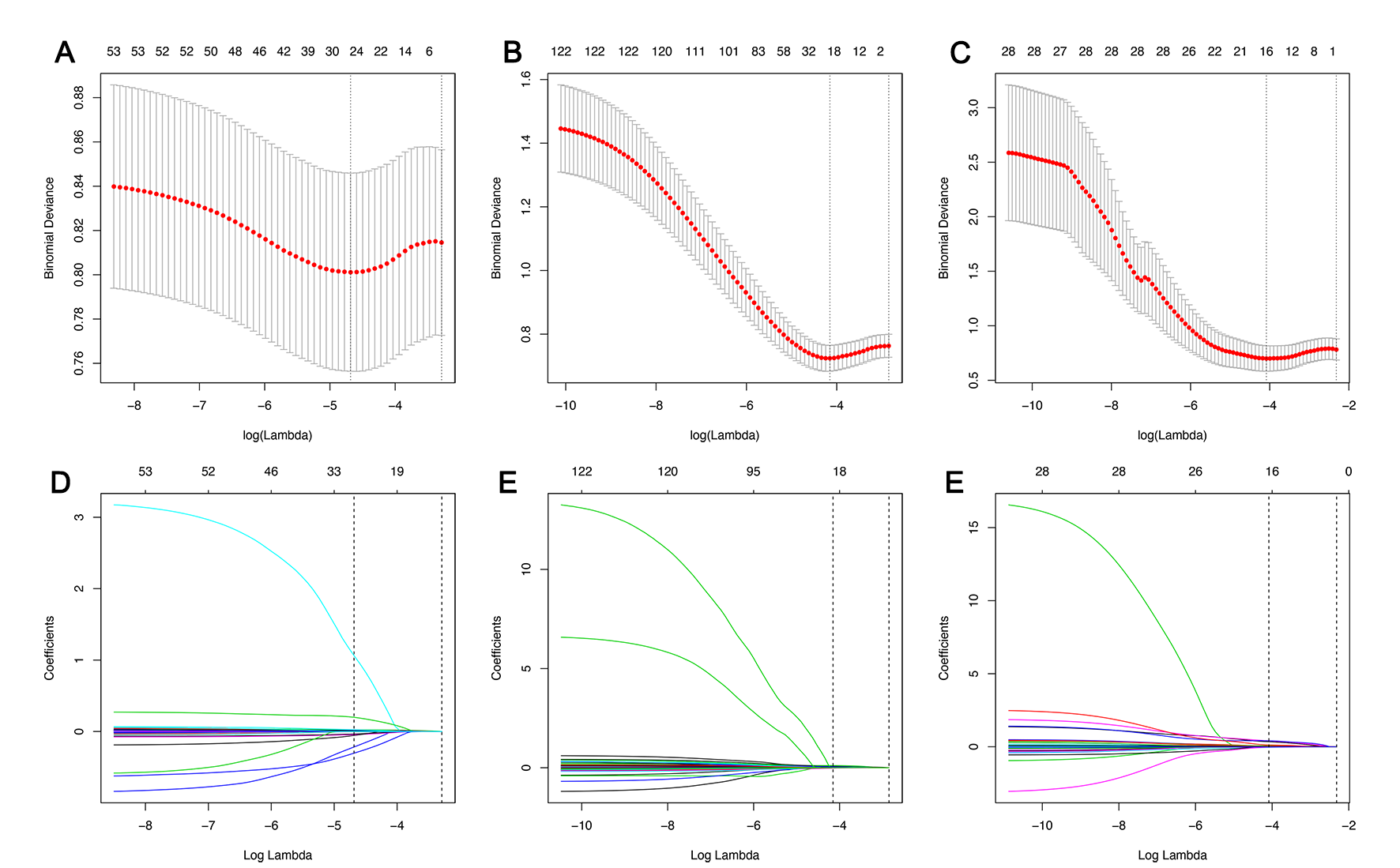

Supplement: Supplementary file 3 [file Image1.tif]
