# Supplementary material for: Comprehensive analysis of liquid-liquid phase separation-related genes in prediction of breast cancer prognosis
Source: Front Genet. 2022 Sep 28;13:834471. doi: 10.3389/fgene.2022.834471 (PMC9554098; doi:10.3389/fgene.2022.834471)
Supplement: Supplementary file 4 [file Table2.docx]

| **Item** | | | **Univariate Cox Analysis** | | | **Multivariate Cox Analysis** | | |
| --- | --- | --- | --- | --- | --- | --- | --- | --- |
|  |  |  | **HR** | **95% Cl** | ***P* value** | **HR** | **95% Cl** | ***P* value** |
| All | Age* | < 45 |  |  |  |  |  |  |
|  |  | 45~64 | 1.078 | 0.650-1.788 | 0.771 | 1.359 | 0.813-2.269 | 0.242 |
|  |  | > 64 | 2.354 | 1.406-3.943 | 0.001 | 2.884 | 1.702-4.886 | <0.001 |
|  | TMB |  | 0.967 | 0.898-1.042 | 0.380 |  |  |  |
|  | Clinical stage* | I~II |  |  |  |  | - |  |
|  |  | III~IV | 2.789 | 1.982-3.923 | <0.001 | 2.275 | 1.489-3.476 | <0.001 |
|  | T stage | T1~2 |  |  |  |  |  |  |
|  |  | T3~4 | 1.871 | 1.278-2.741 | 0.001 |  |  |  |
|  | N stage* | N0 |  |  |  |  |  |  |
|  |  | N1~3 | 2.215 | 1.543-3.181 | <0.001 | 1.679 | 1.067-2.643 | 0.025 |
|  | RiskScore* |  | 1.051 | 1.039-1.063 | <0.001 | 1.051 | 1.039-1.063 | <0.001 |
| TNBC | Age* | < 45 |  |  |  |  |  |  |
|  |  | 45~64 | 0.831 | 0.273-2.529 | 0.745 | 3.460 | 0.657-18.226 | 0.143 |
|  |  | > 64 | 1.930 | 0.590-6.320 | 0.277 | 6.664 | 1.272-34.922 | 0.025 |
|  | TMB |  | 0.830 | 0.597-1.153 | 0.267 |  |  |  |
|  | Clinical stage* | I~II |  |  |  |  |  |  |
|  |  | III~IV | 6.284 | 2.555-15.454 | <0.001 | 4.340 | 1.397-13.486 | 0.011 |
|  | T stage | T1~2 |  |  |  |  |  |  |
|  |  | T3~4 | 2.429 | 0.802-7.356 | 0.116 |  |  |  |
|  | N stage | N0 |  |  |  |  |  |  |
|  |  | N1~3 | 4.123 | 1.596-10.649 | 0.003 |  |  |  |
|  | RiskScore* |  | 1.038 | 1.025-1.051 | <0.001 | 1.040 | 1.021-1.059 | <0.001 |
| Luminal | Age* | < 45 |  |  |  |  |  |  |
|  |  | 45~64 | 0.886 | 0.445-1.765 | 0.732 | 1.054 | 0.520-2.135 | 0.885 |
|  |  | > 64 | 2.276 | 1.159-4.470 | 0.017 | 2.639 | 1.308-5.232 | 0.007 |
|  | TMB |  | 1.006 | 0.959-1.055 | 0.804 |  |  |  |
|  | Clinical stage* | I~II |  |  |  |  |  |  |
|  |  | III~IV | 2.289 | 1.467-3.571 | <0.001 | 2.340 | 1.471-3.720 | <0.001 |
|  | T stage | T1~2 |  |  |  |  |  |  |
|  |  | T3~4 | 1.643 | 0.995-2.714 | 0.053 |  |  |  |
|  | N stage | N0 |  |  |  |  |  |  |
|  |  | N1~3 | 1.881 | 1.183-2.991 | 0.008 |  |  |  |
|  | RiskScore* |  | 1.104 | 1.081-1.128 | <0.001 | 1.096 | 1.071-1.121 | <0.001 |

Table 2: Univariate and multivariate cox regression.
