# Supplementary material for: Comprehensive analysis of liquid-liquid phase separation-related genes in prediction of breast cancer prognosis
Source: Front Genet. 2022 Sep 28;13:834471. doi: 10.3389/fgene.2022.834471 (PMC9554098; doi:10.3389/fgene.2022.834471)
Supplement: Supplementary file 5 [file Table3.docx]

| **Item** | **C-index** | **Lower value** | **Upper value** |
| --- | --- | --- | --- |
| All cohort | 0.784 | 0.741 | 0.827 |
| Luminal cohort | 0.803 | 0.756 | 0.850 |
| TNBC cohort | 0.847 | 0.759 | 0.934 |
